# Mental Health of Students at Polish Universities after Two Years of the Outbreak of COVID-19

**DOI:** 10.3390/ijerph20031921

**Published:** 2023-01-20

**Authors:** Monika Talarowska, Kinga Rucka, Mateusz Kowalczyk, Jan Chodkiewicz, Edward Kowalczyk, Michał Seweryn Karbownik, Monika Sienkiewicz

**Affiliations:** 1 Department of Clinical Psychology and Psychopathology, Institute of Psychology, University of Lodz, al. Rodziny Scheiblerów 2, 90-128 Lodz, Poland; 2 Babinski Memorial Hospital, ul. Aleksandrowska 159, 91-229 Lodz, Poland; 3 Department of Pharmacology and Toxicology, Medical University of Lodz, ul. Żeligowskiego 7/9, 90-752 Lodz, Poland; 4 Department of Pharmaceutical Microbiology and Microbiological Diagnostic, Medical University of Lodz, ul. Muszyńskiego 1, 90-151 Lodz, Poland

**Keywords:** COVID-19, young adults, students, PSS-10, GHQ-28, Mini-COPE

## Abstract

Background: Mental health deterioration in young adults in the aftermath of the COVID-19 pandemic is being increasingly studied. It is clear that the psychological consequences of the pandemic will be evident for many years, especially among the younger generation, who did not have time to acquire adaptive coping strategies before the outbreak of COVID-19. The purpose of this study was to assess the condition of the mental health of students at Polish universities after two years of the pandemic. The types of coping strategies used by the respondents to deal with stress were also evaluated in order to establish which of them could have a beneficial effect on the psyche of young people. Methods: This study included 721 participants (age [years]: M = 25.7, SD = 5.3; 269 (37.2%) males) recruited using snowball sampling from students at two universities in Lodz, Poland, and full-time doctoral students from across Poland (phase I of the study was conducted in March 2019 (*N* = 352); phase II of the study was conducted in April 2022 (*N* = 369)). The following tools were used in this study: The General Health Questionnaire (GHQ-28) by D. Goldberg, the Perceived Stress Scale (PSS-10), and the Inventory for Measuring Coping with Stress (Mini-COPE) by Carver et al. Pearson’s chi-square test and multivariate logistic regression were used in the statistical analysis. Results: The results detailing the condition of the mental health of the subjects, as measured using GHQ-28, were significantly worse in the group surveyed after two years of the pandemic than the results of the survey conducted in March 2019 (adjusted odds ratio for GHQ-28 ≥ 5: 3.66, 95%CI 2.12–6.30, *p* < 0.001). Statistically significant differences were seen for each of the subscales of the GHQ-28 questionnaire. Most often, the subjects complained of anxiety symptoms and sleep disorders, in addition to somatic symptoms. The risk factors for worsening mental health included female (odds ratio 1.70, 95%CI 1.20–2.40, *p* = 0.003) and professional inactivity (odds ratio 1.55, 95%CI 1.04–2.31, *p* = 0.031). On the other hand, the ages of the people surveyed, their relationship status, whether they had children, or the type of university they attended all proved to be insignificant. The following coping strategies had a positive impact on the mental health of the respondents: positive reframing (*Z* = −2.951; *p* = 0.003) and seeking emotional support (*Z* = −2.351; *p* = 0.019). In contrast, strategies such as self-distraction (*Z* = 2.785; *p* = 0.005), denial (*Z* = 2.948; *p* = 0.003), venting (*Z* = 2.337; *p* = 0.019), self-blame (*Z* = 5.511; *p* < 0.001) and behavioral disengagement (*Z* = 4.004; *p* < 0.001) were associated with poorer mental health among the respondents. Conclusions: 1. Of the students surveyed, 33% reported elevated stress levels after two years of the COVID-19 pandemic. 2. The overall mental health of students at Polish universities, as measured by GHQ-28, was significantly worse in the group evaluated after two years of the COVID-19 pandemic, mainly in respect of anxiety symptoms and sleep disorders. 3. Female gender and professional inactivity appeared to be risk factors for the students’ worsening mental health, which may be an indication of the need for further research and planning of psychotherapeutic interventions.

## 1. Introduction

Stress is an inevitable aspect of human existence, and it accompanies us in each of life’s phases. For many people, starting and following a course of studies in higher education is a major life event, not least because it opens the door to a host of opportunities that lie ahead. It is also a most demanding period, both cognitively and emotionally [1].

On one hand, regardless of the type of studies students undertake, higher education appears to confer individual benefits [2]. On the other hand, the very fact of entering university and the effort required to succeed as a student are already a significant source of stress [3]. Students’ living situations are atypical and are difficult to replicate in other areas of social life. They are treated by those around them as adults, responsible for themselves and in control of their future pathways, but at the same time they are also treated as students with responsibilities for which they receive no direct remuneration [4].

Among the stressors specific to this group, the main ones cited include the overload of study-related responsibilities, intense pressure to perform at the best possible level, competition with peers, and, in some cases, the financial burden of paying for university along with other concerns about the future [5,6].

The deterioration of mental health in young adults in the aftermath of the COVID-19 pandemic is being increasingly studied [7,8,9]. It is clear that the psychological consequences of the pandemic will be evident for many years, especially among the younger generation, who did not have time to acquire adaptive coping strategies before the outbreak of COVID-19 [10,11]. To date, however, little is known about possible strategies for the prevention and/or mitigation of the negative impact of the SARS-CoV-2 pandemic on young people’s mental health [12,13,14]. Nevertheless, this issue appears to be extremely important not only because of its long-standing nature, but also because symptoms of depression and anxiety significantly affect young people’s functioning in a range of social roles [15]. Their efficiency in coping with activities such as household chores is then reduced, as is their productivity and academic performance [16,17], and their close personal relationships and social interactions with peers are also negatively affected [16].

The persistence of symptoms of depression and anxiety among young adults was also highlighted before the outbreak of the COVID-19 pandemic [18,19,20]. As early as 2007, Eisenberg et al. [21] found depressive-anxiety symptoms in nearly 16% of the students surveyed, and two years later Zivin et al. [18] confirmed the existence of these symptoms in as many as 60% of people in the aforementioned study group. In their multicenter meta-analysis (16 study projects, 23,649 participants), Satinsky et al. [22] found that the overall prevalence of clinically significant depressive symptoms was 24%, and the overall prevalence of clinically significant anxiety symptoms was 17% (a sample of 9 studies representing 15,626 doctoral students). In contrast, in a project carried out by the team of Garlow et al. [23], the presence of suicidal thoughts in the period of 4 weeks prior to the survey was reported by as many as 11.1% of students. According to Mortier et al. [24], this rate is higher among first-year students and reaches a figure of close to 33%.

Taking into account the aforementioned correlations, the purpose of the study presented was to assess the mental health status of students at Polish universities after two years of the COVID-19 pandemic (phase II of the study was conducted in April 2022). The types of coping strategies used by the respondents to deal with stress were also evaluated in order to establish which of them can have a beneficial effect on the psyche of young people. In addition, the results obtained were compared with those of the survey conducted in March 2019 (phase I of the study), before the outbreak of the pandemic [25].

## 2. Material and Methods

### 2.1. Study Setting and Study Population

#### 2.1.1. Material

The study included 721 participants (age [years]: M = 25.7, SD = 5.3; 269 (37.2%) males) recruited using snowball sampling from students at two universities in Lodz, Poland, and full-time doctoral students from across Poland.

In order to demonstrate the medium effect size of group comparisons (Cohen’s *d* = 0.5) with a statistical power of 0.8 and statistical significance of 0.05, the minimal sample size was estimated to be 64 in each group, as indicated by G*Power, version 3.1.9.2 [26]. The number of recruited volunteers was not limited to that number, in order to facilitate the performance of subgroup and other analyses.

Each subject provided written informed consent to participation in the study.

The inclusion criteria were as follows: the minimum permitted age of participants was 18; participants were required to be students at one of the Polish universities at the time of the study; participants were to have no active COVID-19 infection at the time of the study; participants’ consent to participation in the study was required.

Exclusion criteria were as follows: being under 18 years of age; not being a student at one of the Polish universities at the time of the study; having a confirmed active COVID-19 infection at the time of the study; declining to consent to participation in the study.

#### 2.1.2. Phase I of the Study: March 2019

A total of 830 questionnaires were distributed. A total of 576 were returned, of which 352 were found complete, which accounted for 63.6% of the response rate. The average age of the subjects in the study group was as follows: M = 28.9, SD = 4.8 (in years).

In the recruitment of study participants, purposeful selection was used (doctoral students from all universities associated with the National Representation of Doctoral Students were invited to take part in the research). Doctoral students from technical universities, medical colleges, and universities participated in the study. The subjects were deemed qualified to participate in the study after giving their written informed consent.

#### 2.1.3. Phase II of the Study: April 2022

A total of 369 people were recruited to participate in phase II of the study (average age: M = 22.6, SD = 3.6 (in years)). They included students at the University of Lodz and the Medical University of Lodz. Owing to the ongoing epidemic situation, the survey was conducted electronically using a Google form.

The socio-demographic characteristics of the study group are shown in Table 1.

Statistically significant differences were found between the study groups in terms of age (*Z* = 20.79, *p* < 0.001), gender (*Chi*^2^ = 15.29, *p* < 0.001), relationship status (*Chi*^2^ = 23.43, *p* < 0.001), having offspring (*Chi*^2^ = 46.68, *p* < 0.001), type of school (*Chi*^2^ = 349.78, *p* < 0.001), and professional activity (*Chi*^2^ = 208.53, *p* < 0.001). No significant differences were found in terms of place of residence.

### 2.2. Methods

#### Test Procedure

The presented study was conducted in two phases:

Phase I—the survey was conducted in March 2019 using paper-and-pencil questionnaires.

Phase II—the survey was conducted in April 2022. Owing to the epidemic situation, the survey was conducted electronically using a Google form.

The study used a questionnaire of the authors’ own design, allowing them to collect socio-demographic data. Additionally, the following questionnaires were used:

a. The General Health Questionnaire (GHQ) developed by D. Goldberg and associates [27], distributed in GHQ-28 version [28]. This questionnaire was used for Phase I and Phase II of the study.

The GHQ-28 questionnaire is used to assess the mental health of adults. The intention of its authors was not to make a thorough psychiatric diagnosis, but to determine the probability, the possibility, and the occurrence rate of developing a mental disorder.

GHQ-28 allows clinicians to select people from a given population who are at risk of developing mental health disorders. The GHQ-28 version has 4 scales, each of which includes 7 items: A: somatic symptoms; B: anxiety and insomnia; C: social dysfunction; D: severe depression. The GHQ-28 questionnaire belongs to the so-called self-reports, in which each item is accompanied by 4 possible responses (Not at all, No more than usual, Rather more than usual, Much more than usual). In the questionnaire, the categories of answers appear in a column layout and are assessed as follows: 0–0–1–1. A value of 5 points was taken as the cut-off point, indicating a potential case [28].

Reliability index: 0.911–0.911 for 4 scales and global score; validity indexes: 59% and 75%

The following tests were used for phase II of the study:

b. Perceived Stress Scale (PSS-10) by Cohen et al.

The scale was created to measure perceived stress [29]. It consists of 10 questions that address subjective feelings about problems, personal events, ways of coping and behaviors. The scale is used to assess the intensity of stress over the past month. Internal consistency was expressed by a Cronbach’s alpha coefficient of 0.86 [30].

c. Inventory for Measuring Coping with Stress (Mini-COPE).

Mini-COPE is the Polish abbreviated version of the COPE inventory, created by C. S. Carver, M. F. Scheier, and J. K. Weintraub [31]. It consists of 28 statements that fall under 14 coping strategies (2 statements in each strategy). The questionnaire is self-reporting in nature. The tool is designed for both sick and healthy people. It is used to measure dispositional coping, or ways of reacting and feeling in situations of severe stress. The inventory distinguishes problem-focused coping (active coping, planning, and seeking instrumental support), emotion-focused behaviors (seeking emotional support, religion, and denial), and less effective strategies such as behavioral disengagement, use of substances, and humor.

The score is calculated separately for each scale by adding the points and then dividing the result by 2. The results of each scale range from 0–3.

Cronbach’s alpha coefficients for each scale range from 0.48 to 0.94. The theoretical accuracy of the Polish adaptation of Mini-COPE was established on the basis of an exploratory factor analysis of the results among adults in the age range 18–60 years (M = 32.2; SD = 12.6) and on the basis of correlations of COPE results with those of other tools [30].

### 2.3. Statistical Analysis

Missing data was excluded, and the analyses were based on complete cases only. The main dependent variable was dichotomous (GHQ-28 ≥ 5 or <5), and its frequency was assessed between the groups by using Pearson’s chi-square test and multivariate logistic regression. In the logistic modeling the following independent variables were included: a dichotomous predictor (phase of the study) and—in the case of the adjusted model—several covariates: age (continuous), gender (dichotomous), marital status (dichotomous), offspring (dichotomous), type of school (dummified to two dichotomous variables), and professional activity (dichotomous). There was one unadjusted and one adjusted (multivariate) logistic model built. In the other analyses, dependent variables (PSS-10 and Mini-COPE scales) were treated as ordinal; as a result, between-group differences were evaluated using the Mann-Whitney *U* test, whereas their associations were evaluated using the Spearman’s rank correlation. In case of the analysis of coping strategies (multiple tested hypotheses), the false discovery rate was controlled at the level of 0.05 using the Benjamini and Hochberg procedure; in other cases, *p*-values below 0.05 were considered statistically significant. The analysis was performed using STATISTICA 13.3 Software (StatSoft, Tulsa, OK, USA) [32].

### 2.4. Bioethics

The approval of the Bioethical Commission of the Medical University of Lodz, number RNN/129/18/KE of 10 April 2018 and RNN/29/22/KE of 8 February 2022, for conducting the research was obtained, and the participants gave consent for their data to be used in the research.

## 3. Results

Table 2 shows the results obtained in GHQ-28 by the participants in the first and second phases of the study.

As the data in Table 2 show, the subjects’ mental health status, as measured by GHQ-28, was significantly worse after two years of the pandemic compared with the results of the 2019 survey.

Statistically significant differences are seen for each of the subscales of GHQ-28: somatic symptoms; anxiety and insomnia; social dysfunctions; and depression. Most often, the subjects complained of anxiety symptoms and sleep disorders, somatic symptoms, and deterioration in daily functioning.

Since the groups from different phases of the study showed differences in socio-demographic characteristics, an additional adjusted logistic regression analysis was performed on the following differing group characteristics: age, gender, marital status, offspring, type of school, professional activity. This analysis confirmed the results presented above, indicating a significant difference in the mental health of the subjects before and after two years of the COVID-19 pandemic (odds ratio 3.66, 95%CI 2.12–6.30, *p* < 0.001). In this analysis, female gender (odds ratio 1.70, 95%CI 1.20–2.40, *p* = 0.003) and professional inactivity (odds ratio 1.55, 95%CI 1.04–2.31, *p* = 0.031) were also risk factors for worsening mental health among the subjects. On the other hand, the age of the people surveyed, their relationship status, whether they had children or the type of university they attended proved to be insignificant. Detailed characteristics of the adjusted logistic model is presented in Table 3.

In the next stage of the statistical analysis, the level of perceived stress and the coping strategies used by the subjects, as well as their impact on the subjects’ mental health, were evaluated. The analysis used data from the phase II survey conducted in April 2022 (*N* = 369). The results of the PSS-10 scale, the Mini-COPE questionnaire and the results of the analysis of correlation of the aforementioned questionnaires with the GHQ-28 scale are shown in Table 4.

Assuming a cut-off point of ≥27 points for the PSS-10 scale [29], elevated stress levels were confirmed in 33% of the students surveyed.

The results demonstrate that the sum score of GHQ-28 correlates significantly with the scale of perceived stress (PSS-10): the higher the level of perceived stress, the worse the subjectively perceived mental health in the study group.

The only coping strategies for stress that had a protective effect on the subjects’ mental health status were those that focused on problem-solving: active coping, planning, and seeking instrumental support. In contrast, the use of such coping strategies as venting, behavioral disengagement, use of substances, and self-distraction negatively affected the mental health of the subjects.

Additional analyses (Mann-Whitney U test) between those who had a total score of at least 5 (the cut-off point) and below on the GHQ-28 test confirmed that the following strategies had a positive impact on the condition of the mental health of the subjects: positive reframing (*Z* = −2.951; *p* = 0.003) and seeking emotional support (*Z* = −2.351; *p* = 0.019). Strategies such as self-distraction (*Z* = 2.785; *p* = 0.005), denial (*Z* = 2.948; *p* = 0.003), venting (*Z* = 2.337; *p* = 0.019), self-blame (*Z* = 5.511; *p* < 0.001), and behavioral disengagement (*Z* = 4.004; *p* < 0.001) were associated with poorer mental health among the respondents.

## 4. Discussion

The results of the presented study confirm the elevated stress levels and the deterioration of the mental health of the surveyed students after two years of the COVID-19 pandemic. These results are in line with studies conducted in other countries.

In a project that included 3,099 participants from the Czech Republic (*N* = 1422) and the Slovak Republic (*N* = 1677), Gavurova et al. [33] observed depressive symptoms in 23.4% of Czech and 19.1% of Slovak students (the study used the Patient Health Questionnaire scale for depressive symptoms, PHQ-9). On the PSS-10 scale, with the cut-off point set at a score ≥ 27 points, as many as 12.9% of Czech students and 9.1% of Slovak students experienced high stress. In addition, increased symptoms of anxiety, depression, and higher levels of perceived stress in the study group were associated with a statistically significantly higher risk of internet addiction (*p* < 0.001). Interestingly, in addition to being away from the family home, on-site classes during the semester were a risk factor for significant mental health deterioration [33].

Gavurova et al. [9] also report that somatic complaints affected 72.2% of the Czech students participating in the study, while anxiety symptoms affected 40.3% of those surveyed. In the group of Slovak students, the values were 69.5% and 34.6%, respectively [9]. The results of the regression analysis conducted among the risk factors for mental deterioration in both study groups indicate female gender, younger age, third degree of study, and study in technical fields (informatics, mathematics, and information and communication technologies (ICT)) [9].

In our study, using the same cut-off point (score ≥ 27 points), up to 33% (*N* = 140) of the students reported elevated stress levels. Anxiety symptoms and somatic complaints also predominated among the reported symptoms. Female gender was also shown to be a risk factor for mental health deterioration in young people. On the other hand, the age of the people surveyed, their relationship status, whether they had children, or the type of university they attended proved to be insignificant.

Moreover, Alsairafi et al. [7] point to risk factors analogous to those mentioned above for depressive symptoms among undergraduate students in the health sciences center. These include female gender and age under 29 (female gender is also cited as a risk factor for depressive symptoms during the COVID-19 pandemic by the authors of a study conducted in Jordan, involving 1156 students [34]). Correlations similar to those cited are also indicated by Matias et al. [35]. The authors assessed the anxiety levels of employees of a public university who had contact with people infected with SARS-CoV-2. Female gender was again among the risk factors for an increase in the level of anxiety experienced, as well as psychological symptoms already present in the subjects before the outbreak of the pandemic [35,36].

In a study by Dyson and Renk [37], gender played a significant role in differentiating students in terms of the stress-management strategies they used. Women were more likely to choose strategies that focused on emotions, whereas men were more eager to select problem-focused coping strategies. However, in a longitudinal 20-year study involving young adults, Pelakanakis et al. [38] showed that the respondents who experienced more stressful life events were significantly more likely to use emotion-focused coping strategies, while presenting increased levels of depressive symptoms.

It should be emphasized that a group of young people who were particularly vulnerable to the emotional consequences of the COVID-19 pandemic were those who were receiving psychiatric treatment before March 2019 [39].

In a meta-analysis prepared by a team led by Dragioti et al. [40] that included 173 publications covering the results of studies conducted during the first wave of the pandemic (February–June 2022), the predominance of symptoms such as anxiety, depression, sleep problems, and suicidal ideation was confirmed, while fear and post-traumatic symptoms were mainly present in the elderly. Similar results to those cited are also presented by Marelli et al. [41] and Valenzuela et al. [42].

Charbonnier et al. also point to interesting results from their research [43]. Their team assessed the mental health of French students four times during the first two years of the COVID-19 pandemic. The cited authors observed that measurements reporting the use of maladaptive coping strategies and the severity of depressive symptoms were significantly higher during periods of lockdown than measurements taken during the temporary lifting of lockdown restrictions. Significantly, depressive symptoms were substantially higher in the study group in the second year of the pandemic compared with those observed at its beginning. In addition, with the passage of time and the advent of subsequent lockdowns, the physical activity levels of young adults decreased significantly, and their alcohol consumption increased [44], as did the tendency to eat less healthily [45]. The results suggest that, owing to the prolonged duration of the pandemic, the subjects’ ability to cope effectively with permanent stressors decreased, and this in turn led to an increase in psychopathological symptoms, mainly in the form of anxiety and depression.

Intriguing results are provided by Bonsaken et al. [46]. In their project, they assessed the mental health of college students (*N* = 354) as compared with the mental health of age- and gender-matched non-students (*N* = 3120). The mental health status of the students, as assessed using the GHQ-12 scale, was significantly worse in the former group. The difference was particularly pronounced in those over 30 years of age.

## 5. Summary

A comparison of the mental functioning of the subjects before and during the COVID-19 pandemic confirms the hypothesis of a deterioration in the subjects’ emotional functioning. The experiencing of elevated levels of stress and significant deterioration in sleep quality among students are indicated by Lukowski et al. [47], whereas Graham and Eloff [48] point to reduced general well-being and a deterioration of subjectively perceived mental health. In a study conducted in Brazil by Demenech et al. [49], attention was paid to a statistically significant increase in the risk of suicide attempts observed among students from 2019 to 2020. This rate went up from 11.3% (2019) to 17.0% (2020/2021) and rose particularly among women and those with lower socioeconomic status.

A further important issue seems to be the explanation of the reasons for the deterioration of the mental state of young adults as a consequence of the COVID-19 pandemic.

It appears necessary to develop a screening procedure to identify students who experience a high number of stressors and who are thus exposed to a high risk of mental health problems.

Interventions focusing on stress management should be tailored to address the unpredictability of stressors and people’s individual preferences for specific coping strategies [50].

The results indicate the need to strengthen stress-management strategies such as focusing on problem-solving and seeking emotional support.

Preventive interventions should be extended to students regardless of their field of study or the type of university they attend.

## 6. Conclusions

1. Of the students surveyed, 33% reported elevated stress levels after two years of the COVID-19 pandemic.
2. The overall mental health of students at Polish universities, as measured by GHQ-28, was significantly worse in the group evaluated after two years of the COVID-19 pan-demic, mainly in terms of anxiety symptoms and sleep disorders.
3. Female gender and professional inactivity appeared to be risk factors for the students’ worsening mental health, which may be an indication for further research and planning of psychotherapeutic interventions.

## 7. Limitations

1. The study was not longitudinal, and there were significant differences in the socio-demographic characteristics of individuals from different phases of the study.
2. Since the groups from different phases of the study showed differences in socio-demographic characteristics, an additional adjusted logistic regression analysis was performed on the following differing group characteristics: age, gender, marital status, offspring, type of school, and professional activity. The analysis confirmed the results presented above, demonstrating a significant difference in the mental health of the subjects before and after two years of the COVID-19 pandemic (odds ratio 3.66, 95%CI 2.12–6.30, *p* < 0.001).
3. Phase II of the study took place shortly after the outbreak of the war in Ukraine, which may also have made an impact on the elevated stress levels in the study group.
4. There was no comparison group composed of young adults who were non-students.

## Figures and Tables

**Table 1:** Socio-demographic characteristics of the study group.

Variable	Whole Group *N* = 721	Phase I of the Study *N* = 352	Phase II of the Study *N* = 369
Gender (*N*, %)			
1. female 2. male	452 (62.8%) 269 (37.2%)	196 (55.5%) 156 (44.5%)	257 (69.7%) 112 (30.3%)
Age (years; *M*, *SD*)			
	25.7 (5.3)	28.9 (4.8)	22.6 (3.6)
Place of residence (*N*, %)			
1. up to 50,000 inhabitants 2. 50,000 to 100,000 inhabitants 3. 100,000 to 250,000 inhabitants 4. over 250,000 inhabitants 5. village	103 (14.3%) 54 (7.5%) 50 (6.9%) 412 (57.1%) 102 (14.1%)	42 (11.9%) 22 (6.3%) 32 (9.1%) 202 (57.4) 54 (15.3%)	61 (16.5%) 32 (8.7%) 18 (4.9%) 210 (56.9%) 48 (13.1%)
Marital status (*N*, %)			
Single Partnership	398 (55.2%) 323 (44.8%)	162 (46.1%) 190 (53.9%)	236 (63.9%) 133 (36.1%)
Offspring			
Yes No	68 (9.4%) 653 (90.6%)	60 (17.1%) 292 (82.9%)	8 (2.2%) 361 (97.8%)
Type of school (*N*, %)			
University Medical University Technical University	292 (40.5%) 302 (41.9%) 127 (17.6%)	206 (58.5%) 27 (7.5%) 119 (33.8%)	86 (23.3%) 275 (74.5%) 8 (2.2%)
Professional activity (*N*, %)			
Yes No	300 (41.7%) 421 (58.4%)	110 (31.3%) 242 (68.7%)	311 (84.3%) 58 (15.7%)

Phase I of the study—before the outbreak of the COVID-19 pandemic; Phase II of the study—after two years of the pandemic; M—mean; SD—standard deviation; *N*—number; %—percentage.

**Table 2:** Number and percentage of the study participants showing 5 and more points in each of the GHQ-28 scales.

	Phase I of the Study *N* = 352 (*N*, %)	Phase II of the Study *N* = 369 (*N*, %)	*Chi* ^2^	*p*
A—somatic symptoms	51 (14.5%)	148 (40.1%)	59.17	<0.001 *
B—anxiety, insomnia	68 (19.3%)	187 (50.7%)	77.51	<0.001 *
C—social dysfunction	54 (15.3%)	117 (31.8%)	27.22	<0.001 *
D—severe depression	17 (4.8%)	87 (23.6%)	55.69	<0.001 *
A + B + C + D	181 (51.4%)	304 (82.4%)	78.45	<0.001 *

Phase I of the study—before the outbreak of the COVID-19 pandemic; Phase II of the study—after two years of the pandemic; *N*—number; %—percentage; *—*p* statistically significant, <0.005.

**Table 3:** Details of the adjusted logistic regression model evaluating the link of phase of the study and mental health worsening. *Chi*^2^(8) = 97.34, *p* < 0.001. Hosmer-Lemeshow *Chi*^2^(8) = 3.40, *p* = 0.91.

Type of Independent Variable	Variable	Wald’s Chi-Square	Odds Ratio		*p*-Value
			Point Estimate	95% CI	
Pred	Phase of the study	21.80	3.66	2.12–6.30	<0.001
1	Age	0.61	1.02	0.97–1.06	0.44
2	Gender (F = 1, M = 0)	9.08	1.70	1.20–2.40	0.003
3	Marital status	0.24	0.92	0.64–1.31	0.63
4	Offspring	0.79	0.75	0.40–1.41	0.37
5a	Type of school (Medical)	0.03	1.05	0.63–1.74	0.85
5b	Type of school (Technical)	0.63	1.20	0.77–1.88	0.43
6	Professional activity	4.68	0.65	0.43–0.96	0.031

Type of variable: Pred—predictor; 1–6—covariates; CI—confidence intervals; F—female; M—male.

**Table 4:** The level of perceived stress, the coping strategies used and their impact on the mental health worsening of the subjects (phase II of the study).

Variable	Descriptive Statistics				GHQ-28 A + B + C + D	
					Spearman’s Rank Correlation	
	M	SD	Me	Quartiles	R	*p*
PSS-10	23.23	7.81	24	18–29	0.753	<0.001 *
	Mini-COPE					
Problem-focused strategies	10.49	3.61	11	8–13	−0.126	0.016 *
Emotion-focused strategies	6.53	3.13	6	4–9	0.05	0.341
Venting	3.29	1.41	3	2–4	0.197	<0.001 *
Self-distraction	3.55	1.48	4	3–4	0.123	<0.001 *
Behavioral disengagement	1.62	1.38	2	0–2	0.339	<0.001 *
Use of substances	1.18	1.65	0	0–2	0.217	<0.001 *
Humor	2.31	1.44	2	1–3	−0.056	0.279

PSS-10—Perceived Stress Scale; Mini-COPE—Inventory for Measuring Coping with Stress; GHQ-28—General Health Questionnaire; M—mean; SD—standard deviation; Me—median; *—*p* statistically significant, using Benjamini-Hochberg procedure (corrected significance level = 0.030; false discovery rate = 0.05).

## Data Availability

The datasets generated and analysed during the current study are available from the corresponding author on reasonable request.
